# Correspondence: Reply to ‘On the bonding in ligand-protected gold clusters’

**DOI:** 10.1038/s41467-017-01294-w

**Published:** 2017-11-07

**Authors:** Wen Wu Xu, Xiao Cheng Zeng, Yi Gao

**Affiliations:** 1 Division of Interfacial Water and Key Laboratory of Interfacial Physics and Technology, Shanghai Institute of Applied Physics, Chinese Academy of Sciences, Shanghai, 201800 China; 2 Department of Chemistry, University of Nebraska-Lincoln, Lincoln, NE 68588 USA; 3 Collaborative Innovation Center of Chemistry for Energy Materials, University of Science and Technology of China, Hefei, Anhui 230026 China; 4 Shanghai Science Research Center, Chinese Academy of Sciences, Shanghai, 201204 China

## Introduction

In the recent Correspondence^1^, Professor Henrik Grönbeck made several comments on the recently developed grand unified model (GUM)^2^, in particular using the ligand-protected gold cluster [Au_25_(SR)_18_]^1−^ as an example. We noted that the latter cluster can be viewed as belonging to a special group of ligand-protected gold clusters that contain one or several icosahedral Au_13_ motifs^3^. For this group of clusters, a secondary block Au_13_(8*e*) has been identified as a more convenient way to describe their structure anatomy and evolution. Another extension of the GUM is the identification of the new elementary block, Au_3_(*μ*_3_-S), with zero valence electron [referred as Au_3_(*μ*_3_-S)(0*e*)] to describe all crystallized ligand-protected gold clusters containing *μ*_3_-S motifs^4^. In this correspondence, first, we discuss the purpose of GUM development in more detail. Next, we briefly discuss the secondary block Au_13_(8*e*) and show new computational results on the stabilities of Au_3_(2*e*) and Au_4_(2*e*) elementary blocks. Then two Au_6_^2+^ clusters are used as the simplest example (a suggestion credited to a reviewer of ref. ^2^) to better explain the GUM and high stability of Au_3_(2*e*), followed by a summary and perspective.

*Purpose of GUM development*: First of all, we reiterate a statement pointed out clearly in the abstract of ref. ^2^, that is, “GUM is a predictive heuristic and may not be necessarily reflective of the actual electronic structure”. In other words, the development of GUM is not intended to describe actual electronic structures or electron re-hybridization within the Au core or between the Au core and ligands at atomic level. Density functional theory (DFT) can already serve that purpose.

GUM is a model to highlight a universal correlation between the number of valence electrons in the Au core and the number of elementary blocks, with consideration of the ligand effect. As a rule of thumb, GUM can be used to describe structure anatomy and evolution of the ligand-protected clusters. When using the GUM, the focus is placed on treating the ligand-protected clusters in a coarse-grained fashion in terms of elementary blocks^2^ or secondary blocks^3^, while the counting of valence electrons is at the elementary block level. As such, the assignments of the 1 valence electron for Au atom in the Au core, 0 valence electron for the PR_3_ ligand, and −1 valence electron for halides are empirical descriptions of electronic structure of the ligand-protected gold clusters. Such a description is not intended to reflect the exact electron distribution at the atomic level because these assignments neglect many details in atomic level electronic structures, such as the *s*–*d* hybridization, spin-orbit effects, ligands’ constraint effects, etc. Nevertheless, these detailed assignments of valence electrons and valence electron counting are widely used as rules of thumbs in general chemistry and hence employed in GUM as well.

In summary, the development of GUM is to introduce a generic rule of thumb—a rule derived after analyzing a “big data” of all 71 clusters available in the literature. In practice, we would like the GUM to be used simply as a rule of thumb, particularly for the design and prediction of new ligand-protected Au clusters. If a new ligand-protected cluster, either designed from theory or determined based on mass spectroscopy or transmission electron microscopy (TEM) experiments, does not satisfy the rule of thumb as described in GUM, the predicted structure of the cluster would be questionable or likely unrealistic for next step crystallization effort.

*A secondary block icosahedral Au*_*13*_ *(8e)*: Among the 71 ligand-protected clusters illustrated in ref. ^2^, there is a special group of ligand-protected gold nanoclusters that all contain one or several icosahedral Au_13_ motifs. For these ligand-protected clusters, according to the electron counting protocols for effective detachment of ligands in GUM, each icosahedral Au_13_ motif can be assigned to have 8*e* valence electrons [Au_13_(8*e*)], as each icosahedral Au_13_ motif can be viewed as packing of four elementary blocks. For example, the Au_13_(8*e*) in [Au_25_(SR)_18_]^1−^ consists of two Au_3_(2*e*) and two Au_4_(2*e*) elementary blocks. Note that this decomposition is not intended to reflect the electronic structure of the Au_13_ core at the atomic level but simply to indicate that the 8*e* valence electrons of Au_13_(8*e*) can be viewed as a sum of four pairs of valence electrons of the four elementary blocks. Au_13_(8*e*) can be also viewed as an electron shell closure species, in analog of that of the stable Ne atom. As such, the Au_13_(8*e*) may be regarded as a secondary block (or coarse-grained block) to constitute the gold cores of the special group of ligand-protected gold clusters with one or several icosahedral Au_13_ motifs.

The introduction of secondary block Au_13_(8*e*) into GUM appears to be a convenient supplement to understand this special group of ligand-protected gold clusters with icosahedral Au_13_ motifs. It can be also exploited for predicting new ligand-protected gold clusters by design (see ref. ^3^ for more detail).

**Fig. 1:**
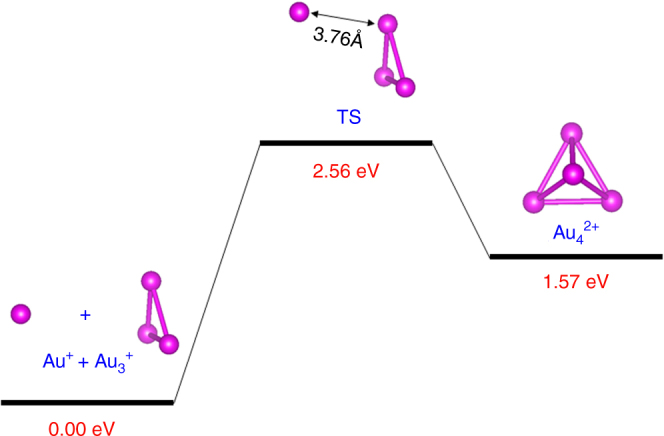
The computed reaction pathway for the Au^+^ + Au_3_^+^ ← → Au_4_^2+^ reaction. Color code: Au—magenta

*Stabilities of Au*_*3*_ *(2e) and Au*_*4*_ *(2e)*: The high stabilities of the trimer Au_3_(2*e*) and tetramer Au_4_(2*e*) elementary blocks are in part due to the strong electronic delocalization among the three- and four-Au atom clusters, and associated strong electron shell closure. From the computed formation energy (Supplementary Table 3 in ref. ^2^), one can see that the Au_4_(2*e*), although not as stable as Au_3_(2*e*), is still highly favorable in formation energy, compared to the isoelectronic dimer Au_2_. In addition, the dissociation barrier from Au_4_^2+^ to Au_3_^+^ and Au^+^ is computed to be ~1 eV (Fig. 1), suggesting the Au_4_^2+^ can be a stable species in the gas phase at room temperature. Thus, Au_3_^+^ and Au_4_^2+^, if could be made in the laboratory, would very likely be a standing-alone/stable species without the ligand protection, largely due to the strong electronic delocalization among Au_3_^+^ and Au_4_^2+^, and associated strong electron shell closure.

**Fig. 2:**
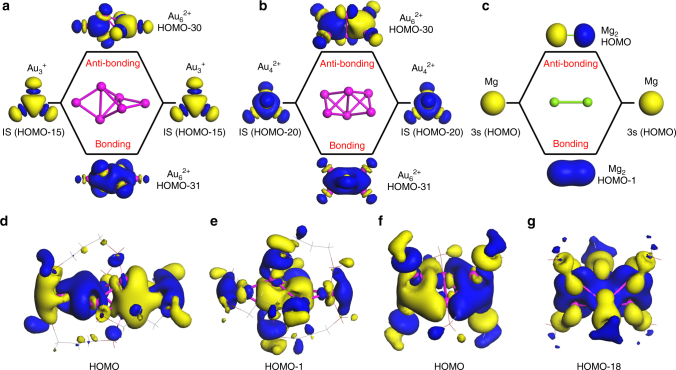
The anti-bonding and bonding orbital diagrams of clusters. **a** *D*_2*d*_ Au_6_^2+^, **b** *D*_2*h*_ Au_6_^2+^, and **c** Mg_2_. The HOMO **d** and HOMO-1 **e** of [Au_6_(dppp)_4_]^2+^, HOMO **f**, and HOMO-18 **g** of [Au_6_(PR_3_)_6_]^2+^. Color code: Au—magenta, Mg—green

*Using Au*_*6*_^*2+*^ *as the simplest example to illustrate GUM*: Here, two Au_6_^2+^ clusters are used as the simplest examples to illustrate the view of the elementary blocks introduced in GUM. The molecular orbital analysis showed that the HOMO-30 and HOMO-31 of the *D*_2*d*_ Au_6_^2+^ core in [Au_6_(dppp)_4_]^2+^ (dppp = 1,3-Bis(diphenylphosphino)propane)^5^ can be viewed as the anti-bonding and bonding orbitals of two HOMO-15 of the Au_3_^+^ cluster (Fig. 2a), respectively, suggesting that the HOMO-30 and HOMO-31 of the *D*_2*d*_ Au_6_^2+^ can be viewed as the linear combination of two 1*S* orbitals of Au_3_(2*e*). Similar behavior can be seen in the HOMO (Fig. 2d) and HOMO-1 (Fig. 2e) of the ligand-protected [Au_6_(dppp)_4_]^2+^. Thus, we use this simple example to demonstrate that the ligand-protected [Au_6_(dppp)_4_]^2+^ and associated *D*_2*d*_ Au_6_^2+^ core can be well described by GUM in terms of the elementary block Au_3_(2*e*).

Likewise, the HOMO-30 and HOMO-31 of the *D*_2*h*_ Au_6_^2+^ core of [Au_6_(PR_3_)_6_]^2+^ ^6^ can be viewed as the anti-bonding and bonding orbitals of two HOMO-20 of Au_4_^2+^ cluster (Fig. 2b), respectively, suggesting that the HOMO-30 and HOMO-31 of the *D*_2*h*_ Au_6_^2+^ can be viewed as a linear combination of two 1*S* orbitals of Au_4_(2*e*). Similar behavior can be seen in the HOMO (Fig. 2f) and HOMO-18 (Fig. 2g) of the ligand-protected [Au_6_(PR_3_)_6_]^2+^. Again, the ligand-protected [Au_6_(PR_3_)_6_]^2+^ and its *D*_2*h*_ Au_6_^2+^ core can be described by GUM as well.

Finally, we note that the orbital analyses on [Au_6_(dppp)_4_]^2+^ and [Au_6_(PR_3_)_6_]^2+^ are quite similar to that on Mg_2_, in which 3*s* orbital (HOMO) of two Mg atoms can form the bonding (HOMO-1) and anti-bonding (HOMO) orbitals of Mg_2_, as shown in Fig. 2c.

*Summary and perspective*: With introducing two groups of elementary blocks in the GUM, Au_3_(2*e*), and Au_4_(2*e*), a variety of ligand-protected gold nanoclusters, e.g., spherical and non-spherical, or magic-number and non-magic-number, can be viewed as an aggregate of the elementary groups by applying electron counting rule, a notion analog to Mingo’s united atom model for understanding weakly bound condensed icosahedra with 8*e* valence electrons for each unit^7^. A recent experiment showed that the localization effect is highly important for the interpretation of the spectroscopy of gold nanoclusters^8^. Moreover, the triangle Au_3_ and tetrahedral Au_4_ can be considered as the basic units of face-centered cubic bulk gold. As such, one may view the ligand-protected gold clusters as the trapped intermediates on the path toward the bulk phase, but being stabilized by the ligands bounded to the surface metal atoms. A similar view has been reported previously for Al and Ga clusters^9^.

In closing, GUM provides a generic, empirical, and coarse-grained model to understand and to assess the structural stabilities and structural evolution of ligand-protected gold clusters, a model that may be extended beyond gold.

## Methods

The reaction pathway for the Au^+^ + Au_3_^+^ ← → Au_4_^2+^ reaction in Fig. 1 was computed using DFT methods with the TPSS functional^10^ and pseudopotential basis set LANL2DZ for Au, as implemented in the Gaussian 09 program package^11^. The orbitals of nanoclusters shown in Fig. 2 were computed based on the DFT method implemented in DMol^3^ ^12,13^. The generalized gradient approximation with the Perdew–Burke–Ernzerhof (PBE)^14^ functional and the double numeric polarized (DNP) basis set coupled with semi-core pseudopotential were employed.

## References

[1] Grönbeck, H. Correspondence: On the bonding in ligand-protected gold clusters. *Nat. Commun.*10.1038/s41467-017-01292-y (2017). 10.1038/s41467-017-01292-y PMC5688090 29142218

[2] Xu WW, Zhu B, Zeng XC, Gao Y (2016). A grand unified model for liganded gold nanoclusters. Nat. Commun..

[3] Xu WW, Zeng XC, Gao Y (2017). Au_13_(8*e*): a secondary block for describing a special group of liganded gold clusters containing icosahedral Au_13_ motifs. Chem. Phys. Lett..

[4] Xu WW, Zeng XC, Gao Y (2017). Au_3_(μ_3_-S)(0e) elementary block: new insights into ligated gold clusters with μ_3_-sulfido motifs. Nanoscale.

[5] Van Der Velden JWA, Bour JJ, Steggerda JJ, Beurskens PT, Roseboom M (1982). Gold clusters. Tetrakis[1,3-bis(diphenylphosphino)propane]hexagold dinitrate: preparation, x-ray analysis, and gold-197 Moessbauer and phosphorus-31{proton} NMR spectra. Inorg. Chem..

[6] Briant, C. E., Hall, K. P., Mingos, D. M. P. & Wheeler, A. C. Synthesis and structural characterisation of hexakis (triphenyl phosphine)-hexagold (2+) nitrate, [Au_6_(PPh_3_)_6_][NO_3_]_2_, and related clusters with edge-sharing bitetrahedral geometries. *J. Chem. Soc. Dalton Trans*. 687–692 (1986).

[7] Mingos DMP (2015). Structural and bonding patterns in gold clusters. Dalton Trans..

[8] Zhou M, Jin R, Sfeir MY, Chen Y, Song Y, Jin R (2017). Electron localization in rod-shaped triicosahedral gold nanocluster. Proc. Natl Acad. Sci. USA.

[9] Schnepf A, Schnӧckel H (2002). Metalloid aluminum and gallium clusters: element modifications on the molecular scale?. Angew. Chem. Int. Ed..

[10] Tao J, Perdew JP, Staroverov VN, Scuseria GE (2003). Climbing the density functional ladder: nonempirical meta-generalized gradient approximation designed for molecules and solids. Phys. Rev. Lett..

[11] Frisch, M. J. et al. *Gaussian 09, Revision B.01* (Gaussian, Inc.: Wallingford, CT, 2010).

[12] Delley B (1990). An all‐electron numerical method for solving the local density functional for polyatomic molecules. J. Chem. Phys..

[13] Delley B (2003). From molecules to solids with the Dmo^l^3 approach. J. Chem. Phys..

[14] Perdew JP, Burke K, Ernzerhof M (1996). Generalized gradient approximation made simple. Phys. Rev. Lett..

